# Evaluating Prostate Cancer: The Diagnostic Impact of MRI and Its Relationship With Transrectal Ultrasound (TRUS)-Guided Biopsy

**DOI:** 10.7759/cureus.69380

**Published:** 2024-09-13

**Authors:** Mohammed Musheer Ahmed, J Kaushik, S Yogesh, Sairam Subburam, Dinesh Raja, Siddarth Thinakaran, MR Madan Karthik Raj, Tejaswee Lohakare, Prashanth A, Gaurav Mittal

**Affiliations:** 1 Surgery, Queen Alexandra Hospital, Portsmouth, GBR; 2 Urology, St. John's Medical College Hospital, Bengaluru, IND; 3 Surgical Oncology, Rajiv Gandhi Cancer Institute and Research Centre, New Delhi, IND; 4 Internal Medicine, Madras Medical College and Rajiv Gandhi Government General Hospital, Chennai, IND; 5 General Medicine, Government Medical College, Omandurar, Chennai, IND; 6 Cardiology, Yerevan State Medical University, Yerevan, ARM; 7 Internal Medicine, SRM Medical College Hospital and Research Centre, Chennai, IND; 8 General Surgery, Vinayaka Mission’s Kirupananda Variyar Medical College and Hospitals, Salem, IND; 9 Child Health Nursing, Smt. Radhikabai Meghe Memorial College of Nursing, Wardha, IND; 10 Physiology, Mahatma Gandhi Institute of Medical Sciences, Wardha, IND; 11 Internal Medicine, Mahatma Gandhi Institute of Medical Sciences, Wardha, IND; 12 Research and Development, Students Network Organization, Mumbai, IND

**Keywords:** benign prostatic hyperplasia (bph), diffusion-weighted imaging (dwi), histopathology, magnetic resonance imaging (mri), multi-parametric mri (mpmri), prostate cancer, transrectal ultrasound (trus), trus-guided biopsy

## Abstract

Background

Prostate disorders, including benign enlargement and malignancy, are commonly evaluated through imaging techniques. Historically, transrectal ultrasound (TRUS) has been used for prostate imaging and biopsy. However, multiparametric MRI (mpMRI), which integrates structural and functional imaging methods, offers enhanced diagnostic capabilities. This study evaluates the effectiveness of mpMRI, including its grading via Prostate Imaging - Reporting and Data System (PI-RADS) or Likert scoring, in distinguishing between benign and malignant prostatic conditions and compares these findings with TRUS outcomes.

Methodology

This prospective study enrolled 30 male patients aged 45 to 75 years (mean age 60 years), selected based on prostatic abnormalities, elevated prostate-specific antigen (PSA) levels (>4 ng/dL), or palpable nodules detected via digital rectal examination. MRI, including PI-RADS or Likert scoring, was utilized to assess prostatic lesions, and results were compared with histopathological data obtained from TRUS-guided biopsies.

Results

Among the 30 patients, common symptoms included urinary retention (60%) and painful urination (53.3%). Malignant tumors were diagnosed in 12 patients (40%). MRI identified eight cases with enlarged transitional zones and irregular signals in peripheral zones (benign prostatic hyperplasia with tumor) and four cases with irregular signals in both zones (sarcoma). Concordance between MRI T2-weighted (T2W) observations and biopsy results showed 60% malignancy detection. Sensitivity assessments revealed MRI detected 15 true-positives (50%), TRUS detected six true positives (20%), and multivoxel spectroscopic analysis (MVS) identified 14 true-positives (46.7%). PI-RADS or Likert scoring of mpMRI was correlated with TRUS outcomes, highlighting its enhanced diagnostic accuracy compared to TRUS alone.

Conclusion

While TRUS remains a standard diagnostic tool, it is limited by significant sampling errors and complications. The integration of mpMRI, with its grading system, significantly improves diagnostic accuracy and treatment planning. Although mpMRI alone has limitations, its combination with contrast-enhanced MRI, diffusion-weighted imaging, and MR spectroscopy offers a comprehensive approach to enhanced prostate cancer detection.

## Introduction

The prostate is a secretory organ comprising both glandular and non-glandular components, located beneath the bladder and surrounding the bladder neck and urethra. Prostatic diseases, including prostatitis, benign prostatic hyperplasia (BPH), and prostate cancer, are prevalent and contribute significantly to morbidity and mortality among men globally [1,2]. The most frequently encountered prostate diseases such as prostatitis, benign prostatic hyperplasia (BPH), and prostate cancer are significant urological conditions that commonly affect the male population, particularly as they age [3]. Prostate carcinoma, in particular, is a major cause of cancer mortality in elderly males [4].

Historically, transrectal ultrasonography (TRUS) has been the primary imaging technique for evaluating prostate conditions. Since its development in 1990, TRUS has been extensively used for screening, diagnosis, and biopsy guidance for both benign and malignant prostatic conditions [5]. Despite its widespread application, TRUS has limitations in terms of specificity and accuracy. In recent years, multiparametric magnetic resonance imaging (mpMRI) has gained prominence as an advanced imaging modality for prostate cancer detection. MpMRI integrates anatomical T2-weighted, T1-weighted, diffusion-weighted, and dynamic contrast-enhanced imaging, providing a comprehensive view of the prostate [6,7]. This integrated approach enhances the differentiation between benign and malignant lesions, offering structural, physiological, and dynamic data that TRUS cannot provide [8].

While TRUS-guided biopsy remains a standard procedure, its accuracy is often compromised by sampling errors and its limited ability to visualize prostatic abnormalities comprehensively. The adoption of mpMRI in clinical practice aims to address these limitations by offering improved diagnostic precision [9]. The addition of mpMRI into prostate cancer screening and diagnosis protocols has shown promise in detecting clinically significant cancers and guiding biopsy decisions, reflecting a shift toward more accurate and less invasive diagnostic strategies [10,11]. This study evaluates the effectiveness of mpMRI in distinguishing between benign and malignant prostatic conditions, focusing on the grading of lesions using Prostate Imaging - Reporting and Data System (PI-RADS) or Likert scoring and comparing these findings with TRUS outcomes. The integration of mpMRI and TRUS data is critical for improving diagnostic accuracy and patient management in prostate cancer care.

## Materials and methods

Study design and setting

This prospective study included 30 consecutive male participants aged 45 to 75 years, with a mean age of 60 years. Inclusion criteria encompassed individuals with suspected prostatic abnormalities, elevated prostate-specific antigen (PSA) levels above 4 ng/dL, or a palpable mass identified through a digital rectal examination. The research was conducted at a single facility - Vinayaka Mission’s Kirupananda Variyar Medical College and Hospital - from January 2024 to May 2024, where prostatic lesions were evaluated using MRI, using PI-RADS or Likert scoring to grade the severity of findings.

Selection Criteria

The inclusion criteria for the study required men aged 40 years or older who were susceptible to prostate abnormalities and eligible to complete all protocol procedures, with increased PSA levels. Exclusion criteria encompassed individuals with a history of prostate surgery, general MRI contraindications such as metal implants, pacemaker devices, or claustrophobia, renal dysfunction with an estimated GFR <50, and general TRUS contraindications including hemorrhoids and acute painful perianal conditions.

Data sources and variables

The study data were collected from comprehensive medical histories of the participants, including urinary symptoms such as urgency, difficulty starting urine flow, painful urination, frequent urination, nighttime symptoms like urge incontinence, dribbling after urination, complete inability to void, bodily discomfort, and occasional fever, along with issues during sexual activity. Diagnostic procedures included abdominal ultrasound, TRUS color Doppler imaging, PSA assessment, and histopathological examination from TRUS-guided prostate biopsies. For the diagnostic assessment of various prostatic pathologies, mpMRI was performed on all patients using a 16-channel conventional pelvic phased-array coil and a 16-channel 1.5 Tesla MR scanner (GE HDxt, GE HealthCare, Chicago, IL). The mpMRI protocol included T2-weighted imaging, diffusion-weighted imaging (DWI), and dynamic contrast-enhanced (DCE) imaging. PI-RADS or Likert scoring was used to classify the severity of the lesions observed on mpMRI. TRUS and multivoxel spectroscopic analysis (MVS) were also performed, with TRUS using a Samsung Ultrasound System HS50 (Samsung Healthcare Global, Seoul, South Korea).

Statistical analysis

Data were entered into a master dataset using Microsoft Excel (Microsoft, Redmond, WA) and analyzed with IBM SPSS Statistics, version 23 (IBM Corp., Armonk, NY). Descriptive statistics, including frequency analysis, percentage analysis, and mean analysis, was used to characterize categorical and continuous variables. Statistical data were presented as mean ± standard deviation (SD). Additionally, the sensitivity, specificity, and accuracy of mpMRI, TRUS, and MVS were evaluated using PI-RADS/Likert scoring in relation to histopathological outcomes.

## Results

Table 1 describes the age and clinical symptoms observed in patients with urological conditions. The average age of the patients was 60 years, with a standard deviation of 10.2 years, and their ages ranging from 45 to 75 years. Regarding clinical symptoms, 18 patients (60%) experienced urinary retention, 16 patients (53.3%) reported painful urination, 12 patients (40%) had blood in their urine, nine patients (30%) suffered from frequent urination, five patients (16.7%) had blood in their semen, and two patients (6.7%) experienced bone discomfort.

**Table 1:** Clinical symptoms in patients with urological conditions The age of the patients ranged from 45 to 75 years with a mean±SD of 60±10.2 years.

Symptom	Number of Patients (n)	Percentage (%)
Urinary Retention	18	60
Painful Urination	16	53.3
Blood in Urine	12	40
Frequent Urination	9	30
Blood in Semen	5	16.7
Bone Discomfort	2	6.7

Table 2 presents a comparative analysis of mpMRI findings and histopathological outcomes. The table details various pathological classifications and their corresponding MRI characteristics, including PI-RADS/Likert scores, along with the number of cases and their percentages. For cystic formations, which were identified with a PI-RADS/Likert score of 1-2, there were three cases, accounting for 10% of the total. Benign prostatic hyperplasia (BPH) was observed with an expanded central zone and a PI-RADS/Likert score of 2-3 in five cases, representing 16.67% of the total. Malignant tumors were identified in eight cases classified as BPH with tumors and a PI-RADS/Likert score of 4-5, and four cases of sarcoma, also with a PI-RADS/Likert score of 4-5. Tissue infarction, characterized by an expanded transitional zone and a PI-RADS/Likert score of 3-4, was found in two cases, making up 6.67% of the participants. Tissue atrophy, indicated by a normal transitional zone and a PI-RADS/Likert score of 2-3, was present in four cases, constituting 13.33% of the subjects. Granulomatous inflammation, with an expanded transitional zone and a PI-RADS/Likert score of 2-3, was also noted in four cases, or 13.33% of the total. The total number of cases was 30, summing to 100%.

**Table 2:** Comparative analysis of mpMRI and histopathological outcomes BPH: Benign prostatic hyperplasia; MRI: magnetic resonance imaging; PI-RADS: Prostate Imaging - Reporting and Data System

Pathological Classification	MRI Characteristics (Including PI-RADS/Likert Scores)	Number of Cases (n)	Percentage (%)
Cystic Formation	Cystic appearance, PI-RADS/Likert Score 1-2	3	10
Benign Prostatic Hyperplasia (BPH)	Expanded central zone, PI-RADS/Likert Score 2-3	5	16.67
Malignant Tumors	BPH + Tumor, PI-RADS/Likert Score 4-5	8	40
	Sarcoma, PI-RADS/Likert Score 4-5	4	
Tissue Infarction	Expanded transitional zone, PI-RADS/Likert Score 3-4	2	6.67
Tissue Atrophy	Normal transitional zone, PI-RADS/Likert Score 2-3	4	13.33
Granulomatous Inflammation	Expanded transitional zone, PI-RADS/Likert Score 2-3	4	13.33
Total		30	100

Table 3 summarizes the comparative analysis of biopsy results and DWI findings. Among the cases with restricted diffusion, classified with a PI-RADS/Likert score of 4-5, 14 were diagnosed with adenocarcinoma, while three cases with facilitated diffusion, having a PI-RADS/Likert score of 1-3, were not diagnosed with adenocarcinoma. Thus, a total of 17 cases were identified through biopsy as having adenocarcinoma.

**Table 3:** Comparative analysis of biopsy results and DWI findings DWI: Diffusion-weighted imaging; PI-RADS: Prostate Imaging - Reporting and Data System

DWI Findings	Biopsy Diagnosis
	Adenocarcinoma
Restricted Diffusion (PI-RADS/Likert 4-5)	14
Facilitated Diffusion (PI-RADS/Likert 1-3)	3
Total	17

Table 4 illustrates the concordance between tissue sampling (considered the gold standard) and MRI T2-weighted imaging observations. It shows the number of malignant and benign cases identified through MRI T2-weighted imaging and biopsy results. Of the lesions detected with abnormal tissue, 18 cases (60%) were categorized as malignant with a PI-RADS score of 4-5, while five cases (16.67%) were classified as benign with a PI-RADS score of 1-3. There were 23 total cases (76.67%) where lesions were detected. Conversely, for cases where no lesion was detected (normal tissue), three cases (10%) were classified as malignant with a PI-RADS score of 4-5, and four cases (13.33%) were classified as benign with a PI-RADS score of 1-3. This resulted in seven cases (23.33%) in which no lesions were identified. In summary, out of 30 cases, 21 (70%) were found to be malignant and nine (30%) were benign.

**Table 4:** Concordance between tissue sampling (gold standard) and MRI T2-weighted imaging observations PI-RADS: Prostate Imaging - Reporting and Data System

MRI T2-weighted observations	Biopsy Results	Malignant	Benign	Total Cases
Lesion Detected	Abnormal Tissue	18 (60%) (PI-RADS 4-5)	5 (16.67%) (PI-RADS 1-3)	23 (76.67%)
No Lesion Detected	Normal Tissue	3 (10%) (PI-RADS 4-5)	4 (13.33%) (PI-RADS 1-3)	7 (23.33%)
Total		21 (70%)	9 (30%)	30 (100%)

Table 5 presents a sensitivity assessment of three diagnostic methods: TRUS, MRI, and multivoxel spectroscopic analysis (MVS). For MRI, out of a total of 30 cases, 15 were identified as true-positives, which constitutes 50% of the cases. However, MRI had seven false-positives, representing 23.3% of the total cases, and eight false-negatives, accounting for 26.7%. In the case of TRUS, it identified six true-positives (20%) and eight true-negatives (26.7%) out of 30 cases. TRUS had nine false-positives, making up 30% of the total cases, and seven false-negatives, which is 23.3%. MVS showed 14 true-positives (46.7%) and six true-negatives (20%) among 30 cases. It had 4 false-positives (13.3%) and six false-negatives (20%).

**Table 5:** Sensitivity Assessment of TRUS, MRI, and Multivoxel Spectroscopic Analysis (MVS) MRI: Magnetic resonance imaging; TRUS: transrectal ultrasound; MVS: multivoxel spectroscopic analysis

Diagnostic Method	Actual Positives (count, percentage)	Actual Negatives (count, percentage)	Incorrect Positives (count, percentage)	Incorrect Negatives (count, percentage)	Total Cases
MRI	15 (50%)	0	7 (23.3%)	8 (26.7%)	30
TRUS	6 (20%)	8 (26.7%)	9 (30%)	7 (23.3%)	30
MVS	14 (46.7%)	6 (20%)	4 (13.3%)	6 (20%)	30

## Discussion

Prostate cancer remains a leading cause of cancer-related mortality among men in developed nations. Effective early detection is crucial for managing the disease, but it continues to present significant challenges [12]. The present study's findings regarding the average age of patients (60 years) and their symptoms align with those reported by Yuen et al. [13], who also observed similar age demographics and clinical symptoms in patients with prostate conditions. The prevalence of symptoms such as urinary retention in 18 patients (60%) and painful urination in 16 patients (53.3%) corroborates earlier research, emphasizing the commonality of these symptoms in prostate pathology. The comparative analysis of MRI findings and histopathological outcomes revealed that malignant tumors were identified in 12 patients (40%). This result is consistent with studies by Wu et al. [14] and Yuen et al. [13], who also reported a significant proportion of malignant findings using MRI. The detection of cystic formations in three patients (10%), benign prostatic hyperplasia (BPH) in five patients (16.67%), and granulomatous inflammation in four patients (13.33%) further supports the findings of these studies, highlighting MRI's utility in identifying various prostate conditions.

The results of the comparison between DWI and biopsy showed that among patients with restricted diffusion, 14 had adenocarcinoma, two had BPH, three had metastasis, and one had lymphoma. This distribution is consistent with the work of Zangos et al. [15] and Beyersdorf et al. [16], who demonstrated the effectiveness of DWI in distinguishing between malignant and benign lesions. Concordance between MRI T2-weighted observations and biopsy results showed that MRI detected malignant lesions in 18 cases (60%), with similar results reported by Fütterer et al. [17] and Ohori et al. [18]. These studies support the reliability of MRI in detecting abnormal tissue, though they also highlight its limitations, such as variability in sensitivity (50% in this study) and specificity. The findings emphasize the importance of incorporating advanced imaging techniques like mpMRI, which includes PI-RADS or Likert scoring systems, in the diagnostic pathway. These systems have been widely adopted in developed countries to guide biopsies and improve the detection of clinically significant prostate cancers. By stratifying lesions based on risk, PI-RADS/Likert scoring enhances the specificity of mpMRI, thereby reducing unnecessary biopsies and improving patient outcomes [17,18].

Moreover, the shift from TRUS to LATP (local anesthesia transperineal prostate biopsy) in clinical practice is largely driven by LATP's superior sampling accuracy and reduced risk of complications. LATP is increasingly favored for its ability to better target anterior lesions and reduce the likelihood of sepsis, a known complication of TRUS [19]. This evolution in practice highlights the need for continued innovation and adoption of more accurate and safer diagnostic techniques in prostate cancer management. Concordance between MRI T2-weighted observations and biopsy results showed that MRI detected malignant lesions in 18 cases (60%), with similar results reported by Fütterer et al. [17] and Ohori et al. [18]. These studies support the reliability of MRI in detecting abnormal tissue, though they also highlight its limitations, such as variability in sensitivity (50% in this study) and specificity.

Sensitivity assessments for MRI, TRUS, and MVS revealed that MRI had a sensitivity of 50%, while TRUS showed a sensitivity of 20%. These findings are in line with the results reported by Fütterer et al. [17] and Ohori et al. [18], who noted the higher sensitivity of MRI compared to TRUS. MVS, with a sensitivity of 46.7%, also reflects the potential value of advanced imaging techniques. The findings of this study are consistent with previous research by Maričić et al. [19], Djavan et al. [20], and Mozer et al. [21], which emphasizes the importance of combining multiple diagnostic approaches to enhance the accuracy of prostate cancer detection. The results support the continued refinement of imaging techniques and the integration of various methods to improve diagnostic precision and patient outcomes, as further supported by Mozer et al. [21], Peltier et al. [22], Sharma et al. [23], and the meta-analysis findings of 2015 [24].

Limitations of the study

This prospective study has several limitations that should be noted. Firstly, the sample size of 30 patients may be inadequate for generalizing the findings to a broader population. Conducting the study at a single center could also restrict the applicability of the results to different clinical settings or patient demographics. While MRI and TRUS are valuable diagnostic tools, they may not fully encompass the variability in prostate cancer presentation and progression. Additionally, the potential for selection bias and the constraints of a single-center study could impact the accuracy and generalizability of the findings. Moreover, the study did not include a detailed analysis of PI-RADS or Likert scoring, which is now a standard in mpMRI for guiding biopsies. Future research involving larger, multi-center cohorts and integrating advanced imaging techniques like LATP and PI-RADS/Likert scoring is necessary to confirm and expand upon these results.

## Conclusions

In conclusion, while transrectal ultrasound (TRUS) biopsy remains the standard for diagnosing prostate cancer, it is prone to significant sampling errors, potentially missing a large number of cancers and underestimating tumor severity, especially in anterior lesions. This method also carries a higher risk of complications. MRI enhances diagnostic safety and assists in treatment planning and staging. Although T2-weighted MRI provides excellent soft tissue imaging, its ability to detect and localize prostate cancer is limited. The amalgamation of dynamic contrast-enhanced magnetic resonance imaging, diffusion-weighted imaging, multiparametric MR imaging, and magnetic resonance spectroscopy markedly improves diagnostic accuracy, offering a more comprehensive approach to prostate cancer assessment. Additionally, the adoption of PI-RADS/Likert scoring in mpMRI and the shift toward LATP are promising advancements that could further refine prostate cancer diagnosis and management, ultimately improving patient outcomes.
